# Physician recruitment and retention in New Brunswick: a medical student perspective

**Published:** 2016-10-18

**Authors:** Mariah Giberson, Joshua Murray, Edward Percy

**Affiliations:** 1 Faculty of Medicine, Dalhousie Medicine New Brunswick, Saint John, NB; 2 Horizon Health Network, Moncton, NB

## Abstract

**Background:**

Physician recruitment and retention is a priority for many Canadian provinces. Each province is unique in terms of recruitment strategies and packages offered; however, little is known about how medical students evaluate these programs. The purpose of the current study was to determine which factors matter most to New Brunswick (NB) medical students when considering their location of future practice.

**Method:**

A survey of NB medical students was conducted. Descriptive statistics were produced and a linear regression model was developed to study factors predictive of a student’s expressed willingness to practice in NB.

**Results:**

158 medical students completed the online survey, which is a response rate of 55%. Job availability and spouse’s ability to work in the province were ranked as the top factors in deciding where to practice. In the final regression model, factors predictive of an expressed desire to practice in NB include being female, living in NB prior to medical school, attending medical school at Université de Sherbrooke, participation in the NB Preceptorship program, and a desire to practice family medicine.

**Conclusions:**

This study provides insight into what medical students consider when deciding where to practice. This research may be used to inform physician recruitment efforts and guide future research into medical education and policy.

## Introduction

Physician recruitment has long been a concern of provincial governments, Canadian medical schools and patients. It is of particular concern in rural communities, which often have more difficulty attracting physicians. Given the challenge of attracting physicians to rural areas, provinces and communities across Canada have developed incentive packages and programs to aid in recruitment. Many provinces offer return-of-service agreements that provide bursaries to medical students or physicians in exchange for a commitment of time to practice in an underserviced area. 93.9% of rural New Brunswickers have a family physician compared to 90.9% of the urban population.^1^ The New Brunswick (NB) government offers one of the more modest incentive packages: $20,000 to family practitioners and “hard to recruit” specialists who sign a mandatory two-year return-of-service agreement to work in a community outside the major cities.^2^ The most populous metropolitan area in New Brunswick has a population of only 138,000.^3^ The province also supports a Summer Preceptorship program for NB medical students, wherein students are paid an hourly wage to job shadow physicians in the province. This program is designed to expose medical students to the scope of medical practice in NB.

Given the high cost of attending medical school rising, these financial incentives can be appealing to a debt-burdened student. While these programs work well in the short term, proof of their long-term effectiveness is lacking.^4^ One study of Newfoundland’s return-of-service program showed 72% of physicians with agreements fulfilled their obligation.^5^ A study by Sempowski found that return of service agreements were not successful for the long-term retention of rural family physicians.^4^

Within the Canadian medical community there has been a push to strategize hiring for physicians across the country. Research is lacking, however, when it comes to predicting what factors influence a physician’s choice. One study conducted in Alberta found that spousal influence, type of practice and proximity to extended family were the factors that most influenced current practice locations.^6^ An Australian study found similar results: physicians rated family, spousal and community factors higher than economic ones such as loan forgiveness and signing bonuses when deciding where to practice.^6^,^7^ One attempt at addressing these factors has been to train medical students in their home province. New Brunswick has recently opened Dalhousie Medicine New Brunswick (DMNB); a satellite campus of Dalhousie Medicine and the province’s first English-speaking medical school. All NB citizens accepted into Dalhousie medicine must study at the DMNB satellite campus in Saint John. A study done by Université de Sherbrooke, a francophone medical school with a satellite campus in Moncton New Brunswick, examined the effects of the length, timing and frequency of exposure to the NB on likelihood that a doctor will return work in the province. Similar to Dalhousie’s structure, NB citizens must study at the Université de Sherbrooke satellite campus in Moncton-referred to as Sherbrooke in this paper. The study found that the longer students were exposed to working in NB during their medical training, the more likely they were to be practicing in the province at the time of the study. Family doctors who spent time in their home province of NB in all four years of undergraduate medical training were 9.3 times more likely to practice in NB compared to their counterparts who had no expose to NB during medical school.^8^

No literature could be found that directly measured factors important in the decision of where to practice for New Brunswick physicians or medical students. This represents a gap in the current knowledge of physician recruitment. This purpose of this study was to survey medical students whose home province is New Brunswick in order to determine the factors most important to them when deciding where to practice in the future and to determine which factors correlated with their willingness to practice in the province.

## Methods

### Participants

The study population was 287 New Brunswick medical students, as listed with the NB Department of Health. Participants had to graduate medical school between 2014 and 2017. Participants were excluded from the study if they had never lived in NB. For the purposes of this study, NB medical student is defined as a medical student who lived in NB prior to medical school.

### Survey

A pilot study was conducted via focus group interviews of medical students from Dalhousie and Memorial University who identified NB as their home province. Each year Memorial has 10 positions available for NB citizens. The responses identified were used to guide the development of the questions included in the study’s survey.

The survey was hosted through the Opinio web-based survey software program. Access to the survey and survey answers were password protected. The program ensured anonymity and only allowed participants to answer the survey once.

The survey was comprised of demographic questions on gender, age, marital status, children, previous residence in NB and/or having attended high school in NB. Data on medical school, expected year of graduation, specialty of choice, and previous participation in the NB Summer Preceptorship program was also collected.

Participants were asked to rate the likelihood that they will seek to practice in New Brunswick on a 10-point scale. Respondents were also asked to rank a series of 12 factors in order of which they considered most important in deciding whether to practice medicine in NB. These factors were: clinical experience in NB, having family/friends in NB, community recreation, spouse/significant other’s ability to work in NB, opportunities for research in NB, desire to have a rural practice, job availability for your chosen specialty in NB, availability of residency positions in NB, financial incentives, return-of-service agreements, perceived relations between physicians and government. The study did not ask where in NB students would like to practice, and considered NB as a whole rather than differentiating between rural and urban.

### Procedure

The survey was sent to 287 medical students listed as NB medical students on a NB Department of Health database. Invitations to participate were sent through email using the Opinio software. The students were assured that participation was voluntary and their responses were confidential. Consent was required before proceeding to the electronic survey.

### Analysis

Descriptive statistics were used to summarize participant demographics. Counts and proportions were used for categorical variables while means and standard deviations were used for continuous variables. The main outcome was the answer to the question: “What is the likelihood that you will practice medicine in New Brunswick?”, which was measured on a 10-point scale.

Basic associations between the main outcome and demographic variables were assessed with the use of *t*-tests and with ANOVA *F*-tests for demographic variables with more than 2 levels. Simple linear regression was used to assess the association between age and the main outcome. Respondents were asked to rank a series of 12 factors (from 1 to 12) that were important in deciding whether to practice medicine in NB. Correlations were assessed to see which of the 12 factors were ranked highly together. Linear regression and diagnostic plots were examined to assess the form and strength of the relationship between these 12 factors and the main outcome. Due to poor linear fit between the factors and the outcome, the 12 factors were dichotomized into whether or not respondents included them in their top 3 choices. Associations between the categorized factors and the main outcome were assessed using *t*-tests.

A multivariate linear regression model was constructed to determine which factors were associated with the likelihood to practice medicine in NB. Initially a series of simple linear regression models were fit for all demographic predictors. Variables that were significant at the *α* = 0.2 level were carried over to the multivariate model fitting stage. A stepwise AIC method was used to select the final multivariate model. Significance was assessed at the *α* = 0.05 level. All analyses were conducted using R version 3.0.2.

## Results

There were 158 completed responses, corresponding to a 55% (158/287) response rate. Respondents were 42.4% (67/158) male and 57.0% (90/158) female, with one participant choosing not to disclose. The mean age of respondents was 25.5 years (*SD*=4.76). Demographic data, along with mean likelihood of practicing in New Brunswick ratings are presented in Table 1.

Medical school, gender, living in NB prior to medical school, participation in the preceptorship program, and specialty of choice were all significantly associated with a perceived likelihood of practicing medicine in New Brunswick. On average, respondents attending l’Université de Sherbrooke rated themselves as more likely to practice in NB than students of other schools (*p* = 0.009). Females ranked their likelihood to practice medicine in NB higher than their male counterparts (*p* = 0.046). Those living in NB prior to starting medical school and those who participated in the NB summer preceptorship program rated higher likelihood as well (*p* < 0.001 and *p* = 0.022, respectively). Planning to choose family medicine was also associated with a higher rated likelihood or practicing in NB (*p* = 0.012). There was not enough evidence to conclude there is a difference in outcome between those who did and did not have children (*p* = 0.056). Year of graduation, relationship status, and graduating from a NB high school were not significant in this study.

Of the twelve factors identified in the pilot study as important factors when deciding where to practice, job availability of the respondent’s desired specialty in NB was ranked most important 22% of the time. The second most important factor was spouse/significant other’s ability to work in NB with 15% of respondents ranking it was the number one factor affecting their decision making. Desire to have a rural practice, opportunities to do research in NB, and attending medical school in NB were most often ranked last. Table 2 shows the relationship between factors important to the residents in deciding to practice medicine in NB and their overall likelihood of practicing medicine in NB.

The final multivariate model included 6 predictor variables: Medical school (Sherbrooke), living in NB prior to medical school, having children, participation in the NB preceptorship program, and having ranked clinical experience in NB and/or financial incentives outside the top 3 factors. These were all associated with a higher reported likelihood of practicing in NB.

The final model was an improvement on the null model (F 9,118 = 4.7, *p* <0.001) and had an *R* of 0.264. Those who attended the Sherbrooke program expressed greater likelihood of practicing in NB than respondents from Dalhousie, and Other schools (meaning not Dalhousie, Memorial or Sherbrooke). The difference between those at Sherbrooke and Memorial was not significant.

Living in NB prior to medical school was associated with outcome scores 1.7 units higher than those who did not (95% CI= 0.41, 3.03, *p*=0.011). Similarly, those with children had outcomes scores 1.8 units higher than those without children (95% CI = 0.17, 3.3, *p* = 0.030).

## Discussion

Medical school, female gender, living in NB prior to medical school, participation in the NB summer preceptorship program and desired specialty were all significantly associated with the expressed likelihood of practicing in NB. In the final regression model, parenthood was significantly associated with the outcome. This could be explained by the fact that children will have ties to the province (school, friends) and this would be an incentive to stay in the province.

Relationship status did not significantly influence the outcome. This was unexpected as spouse’s ability to work in NB was ranked as an important factor when considering to practice in NB. Another study found that spousal influences were more important to female physicians than male physicians when choosing practice location.^6^ Previous studies of family medicine residents found family-related factors had the most influence on practice location, especially for married physicians.^7^ Sixty-two percent of the respondents in this study stated they were either married or in a serious relationship.

Year of graduation did not affect the outcome. When the survey was conducted the class of 2014 had already received their residency placements. This could mean that knowing where one was going for their residency did not significantly change the student’s ranking of likelihood to work in NB. This is also important, as there are a limited number of residency training positions in the province. Living in NB prior to medical school was significantly associated with the outcome, but graduating from an NB high school was not significantly associated with the outcome. This finding may influence medical schools with residential requirements to rethink how they are confirming residence in the province. For some, the only proof required to show that a student is a resident of the province is a mailing address.

Desire to have a rural practice was often ranked in the bottom three factors influencing desire to practice in NB. This lack of enthusiasm for rural practice may be significant as 48% of NB residents are considered to live in a rural area.^9^ The American Academy of Family Physicians guidelines for rural practice recommends implementing rural training during medical school.^10^ The literature suggests that physicians from rural backgrounds are more likely to choose a career in rural medicine compared to urban students.^11^,^12^,^13^ However, exposure to rural practice during medical school and residency can influence urban-raised students to choose a career in rural medicine.^13^

As this is a self-reported survey there is response bias. This study does, however, provide valuable insight into how a large proportion of New Brunswick’s medical students feel about their job prospects in this province and the factors that are important in their decision making process. The New Brunswick Society of Physicians and Surgeons estimates the number of New Brunswickers without a family doctor to be close to 50 000.^14^ The group of students surveyed will likely make up a large portion of the physician workforce in the near future and it is important to study this group from a human resources and planning perspective.

This study provides a snapshot of how the medical students felt at one point in time. The decision for where to practice is likely fluid and thus it would be valuable to do a prospective study of students throughout their training in the future. However, we believe our results may be of value when provinces consider recruitment and retention strategies for physicians in the future. Information presented may also influence medical schools to reconsider how they assess a student’s home province, and how rural experiences can be incorporated into training.

## Figures and Tables

**Table 1:** Likelihood of practicing medicine in New Brunswick as correlated with demographic data

Demographic	n (%)	Likelihood of practicing in NB M (SD)	P-Value
**Medical School**			
Sherbrooke	43 (28.1)	8.05 (1.93)	0.009*
Dalhousie	56 (36.6)	6.63 (2.65)	
Memorial	24 (15.7)	6.35 (2.65)	
Other	30 (19.6)	6.48 (2.65)	

**Year of graduation**			
2014	35 (23.0)	6.57 (2.87)	0.6588
2015	31 (20.4)	6.88 (2.41)	
2016	47 (30.9)	7.26 (2.23)	
2017	39 (25.7)	6.75 (2.35)	

**Gender**			
Female	90 (57.3)	7.24 (2.41)	0.0460*
Male	67 (42.7)	6.39 (2.51)	

**Relationship Status**			
Married	20 (12.7)	7.81 (2.46)	0.2913
Serious relationship	77 (49.0)	6.85 (2.45)	
Single	60 (38.2)	6.75 (2.41)	

**Lived in NB prior to medical school?**			
No	21 (13.5)	4.53 (2.27)	0.000*
Yes	135 (86.5)	7.20 (2.34)	

**NB high school graduate?**			
No	11 (7.0)	7.55 (2.30)	0.3540
Yes	147 (93.0)	6.82 (2.50)	

**Children**			
Children No	150 (94.9)	6.78 (2.49)	0.0564
Children	Yes 8 (5.1)	8.50 (1.77)	

**Participation in Preceptorship Program**			
Yes	82 (59.4)	7.27 (2.39)	
No	39 (28.3)	6.00 (2.54)	0.0220*
No, but I plan to this summer	17 (12.3)	7.29 (2.11)	

**Specialty**			
Family Medicine	43 (34.1)	7.53 (2.65)	0.0115*
Non-surgical specialty	43 (34.1)	6.93 (2.22)	
Surgical specialty	17 (13.5)	5.24 (2.80)	
Unsure	23 (18.3)	7.09 (1.70)	

**Table 2:** Associations between factors that are important when deciding to practice medicine in NB and the likelihood that the respondent would practice medicine in NB

Demographic	Factor ranked in top 3 mean (SD)	Factor ranked outside top 3 mean (SD)	P-Value
Clinical experience	6.36 (2.21)	7.09 (2.47)	0.155
Family/friends in NB	7.45 (2.20)	6.55 (2.56)	0.034*
Community recreation	6.83 (2.92)	6.94 (2.42)	0.891
Spouse/significant other’s ability to work in NB	7.05 (2.38)	6.79 (2.53)	0.541
Opportunity to do research in NB	6.89 (2.79)	6.91 (2.38)	0.977
Desire to have a rural practice	6.91 (2.77)	6.91 (2.35)	0.997
Job availability for our specialty in NB	7.05 (2.39)	6.76 (2.66)	0.507
Attending medical school in NB	6.58 (2.60)	6.97 (2.41)	0.483
Residency availability in NB	7.00 (2.50)	6.87 (2.46)	0.810
Financial incentives	5.93 (2.83)	7.10 (2.32)	0.026*
Return of service agreement	6.97 (2.21)	6.86 (2.54)	0.837
Perceived relations between physicians and NB government	6.50 (2.54)	6.95 (2.46)	0.455
